# Metastatic lobular carcinoma of the breast masquerading as a primary rectal cancer

**DOI:** 10.1186/1477-7819-10-231

**Published:** 2012-10-31

**Authors:** Ikuo Matsuda, Nagahide Matsubara, Nobuo Aoyama, Mie Hamanaka, Daisuke Yamagishi, Takashi Kuno, Kiyoshi Tsukamoto, Tomoki Yamano, Masafumi Noda, Hiroki Ikeuchi, Naohiro Tomita, Seiichi Hirota

**Affiliations:** 1 Department of Surgical Pathology, Hyogo College of Medicine, 1-1 Mukogawa-cho, Nishinomiya, Hyogo, 663-8501, Japan; 2 Department of Surgery, Hyogo College of Medicine, Nishinomiya, 663-8501, Japan; 3 Aoyama Clinic, Kobe, 650-0015, Japan

**Keywords:** Lobular carcinoma, Breast, Rectal metastasis

## Abstract

**Background:**

Colorectal metastasis of lobular carcinoma of the breast is a diagnostic challenge. It may macroscopically simulate primary colon cancer or inflammatory bowel disease. In some cases, the interval between the primary breast cancer and metastatic colorectal lesions is so long that the critical records for diagnosis including history might be lost or missed.

**Case presentation:**

Reported herein is a case of metastatic lobular carcinoma of the breast masquerading as a primary rectal cancer developed in a 62-year-old Japanese woman. The case initially presented as a circumferential rectal lesion, and information on the patient’s history of breast cancer was not noted. As the result of endoscopic biopsy, diagnosis of poorly differentiated rectal adenocarcinoma was made. The lesion was surgically resected after chemo-radiotherapy. Histopathological examination of the resected specimen with hematoxylin and eosin (HE) stain revealed a single-file arrangement of the tumor cells, reminiscent of lobular carcinoma of the breast. Immunohistochemical analysis revealed an immunophenotype consistent with lobular carcinoma of the breast. Because further review of the patient’s history revealed an occurrence of ‘poorly differentiated adenocarcinoma of the breast’, which she had experienced 24 years earlier, the final diagnosis of the lesion was made as rectal metastasis from lobular breast carcinoma.

**Conclusions:**

Poorly differentiated adenocarcinoma of the colorectum is rarer than that of the stomach. Linitis plastica-type cancer of the colorectum is also rarer than that of the stomach. A lesson from the present case is that before we conclude a linitis plastica-type cancer of poorly differentiated type as a primary colorectal cancer, it is critical to exclude a possibility of metastatic colorectal cancer.

## Background

Colorectal metastasis of lobular carcinoma of the breast is a diagnostic challenge. It macroscopically simulates primary colon cancer or inflammatory bowel disease. In some cases, the interval between the primary breast cancer and occurrence of colorectal lesions is so long that the critical history is lost or missed. Reported herein is a case of metastatic lobular carcinoma of the breast masquerading as a primary rectal cancer in a 62-year-old Japanese woman.

## Case presentation

**Figure 1:**
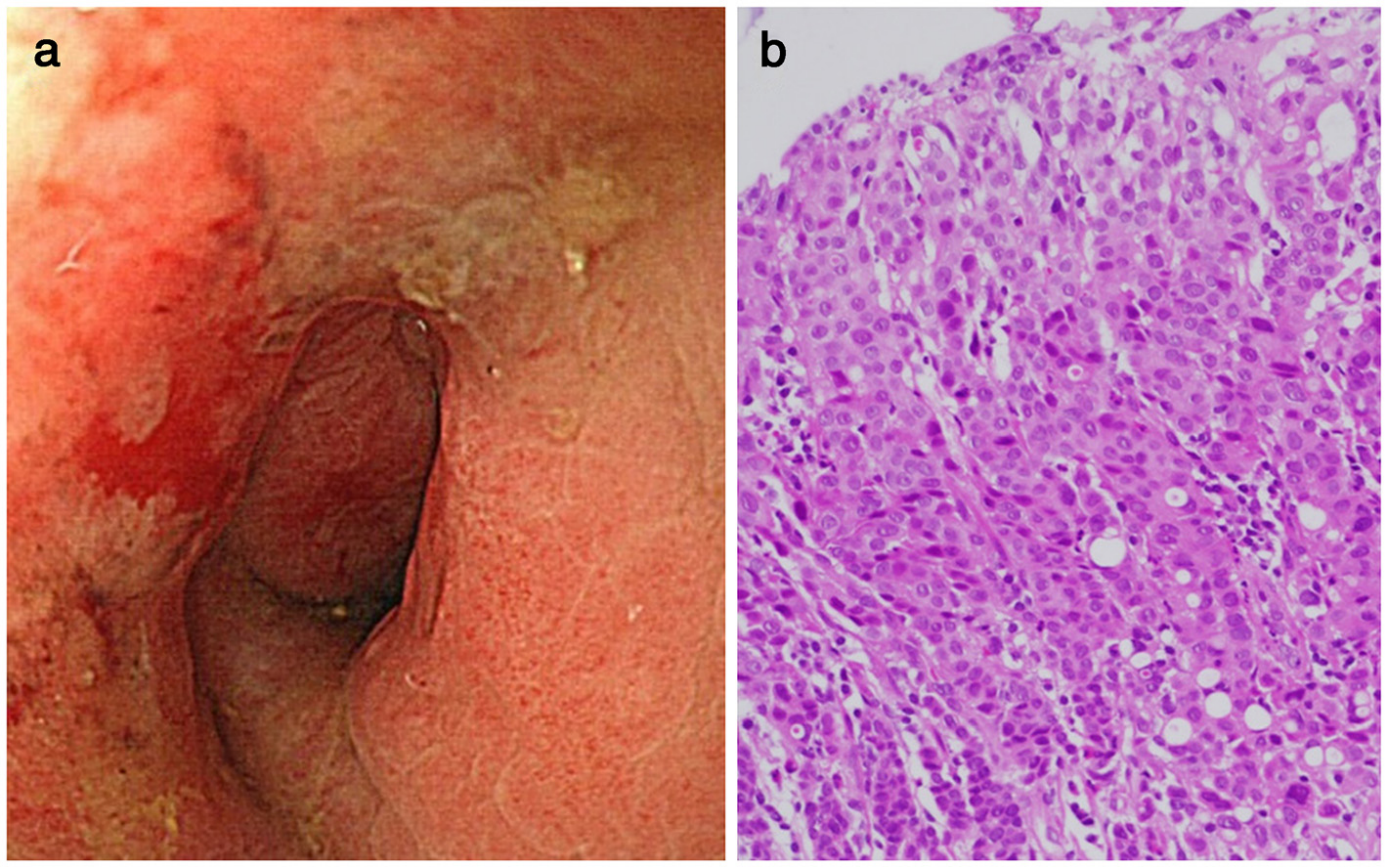
**Preoperative endoscopy of the lesion.** Endoscopy showed circumferential stricture with erosive change of the rectum (**a**), and biopsy specimen from the lesion revealed poorly differentiated adenocarcinoma (**b**) (HE stain, ×400).

**Figure 2:**
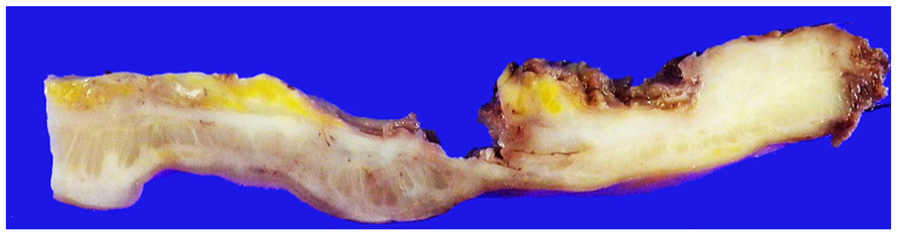
**Cut surface of the resected rectal tumor after formalin fixation.** Macroscopic examination showed a diffusely invasive lesion (in white) with no clear border. Note white trabeculae of the tumor penetrating vertically through the muscularis propria. The upper side was luminal whereas the lower side was serosal.

A 62-year-old Japanese woman was referred to a nearby clinic because of progressive constipation for six months. Endoscopic examination of her colon revealed two circumferential strictures at the ascending colon and rectum (Figure 1a). Hematoxylin and eosin (HE) image of the endoscopic biopsy of the rectal lesion led us to a diagnosis of poorly differentiated adenocarcinoma of the rectum (Figure 1b). The biopsy of the lesion of the ascending colon revealed colitis. Total resection of the tumor was predicted to be difficult due to size and infiltrative border of the tumor. Therefore, the patient was subjected to chemo-radiotherapy. After five courses of the chemo-radiotherapy in total of 45 Gy of radiation plus tegafur gimeracil oteracil potassium (TS-1) and irinotecan (CPT-11) as chemotherapy with minimal clinical response, a proctectomy was performed with low anterior resection. Macroscopically, the rectal mass was a diffusely invasive lesion with no clear border. Cut surface showed white trabeculae penetrating vertically through the muscularis propria (Figure 2).

**Figure 3:**
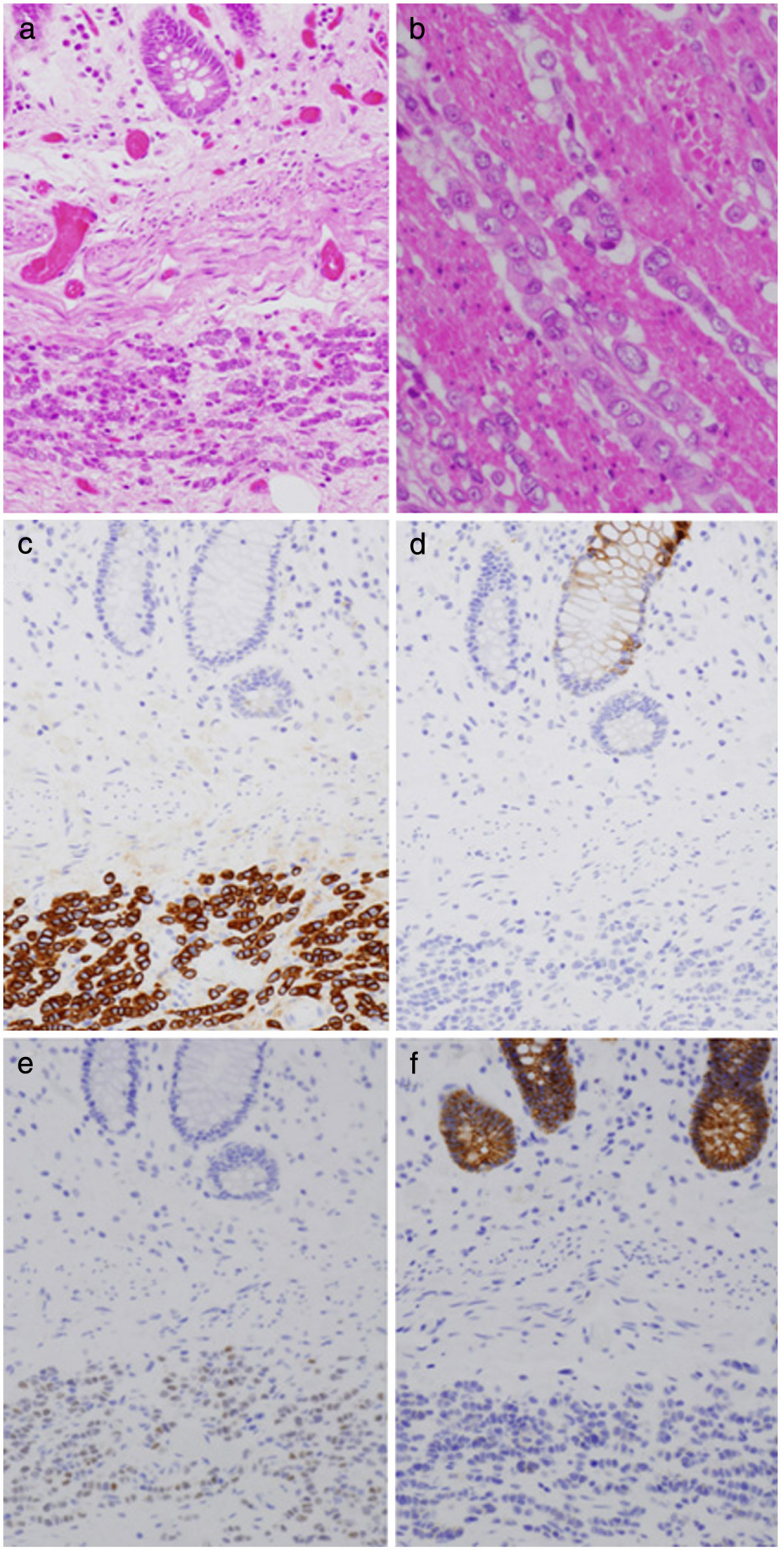
**Histological examination of the resected rectal tumor.** (**a**,**b**) Hematoxylin and eosin (HE) staining showed diffuse and infiltrative proliferation of small tumor cells in the submucosal region (**a**, original magnification ×200) and through the muscularis propria (**b**, original magnification x400). Minimal cohesion was observed among the tumor cells, and each tumor cell had an eccentric nucleus and eosinophilic cytoplasm. (**c**-**f**) Immunohistochemical findings. The tumor cells were positive for CK7 (**c**) but not for CK20 (**d**). They were positive for Estrogen receptor α (**e**) but negative for E-cadherin (**f**). In (**a**) and (**c-f**), the lower half of each figure was occupied by the tumor cell nests in the submucosal region, whereas in the upper half, the background normal colon crypts were observed (original magnification ×200).

Microscopic examination of resected specimen revealed a diffuse and infiltrative proliferation of small tumor cells (Figure 3a,b). There was no apparent degeneration or necrosis of the tumor cells, suggesting the minimal pathological effect of chemo-radiotherapy on the tumor cells. Minimal cohesion was observed among the tumor cells. Of note was the single-file arrangement of the tumor cells (Figure 3b), characteristically observed in lobular carcinoma of the breast, but rarely found in colon cancer. These observations of an HE-stained specimen made us suspect metastatic lobular carcinoma of the breast, instead of primary rectal cancer.

To confirm this, immunohistochemical analysis was performed (Figure 3c-3f). The tumor cells were positively stained for CK7 (Figure 3c) and Estrogen receptor α (Figure 3e), while they were negative for CK20 (Figure 3d) and E-cadherin (Figure 3f). In contrast, the background colon epithelial cells were positive for CK20 (Figure 3d) and E-cadherin (Figure 3f), while they were negative for CK7 (Figure 3c) and estrogen receptor α (Figure 3e).

We reexamined the patient’s history and found that she had undergone surgery for left breast cancer 24 years earlier. The pathological diagnosis of the cancer was found to be ‘poorly differentiated adenocarcinoma, scirrhous type,’ with metastasis in her left axillary lymph nodes. Unfortunately, the glass slides and the paraffin blocks of the breast cancer were lost during the turmoil of the Hanshin-Awaji Earthquake in 1995. Therefore, we could not reconfirm the diagnosis of the primary breast cancer.

We reexamined the endoscopic biopsy of the rectum (Figure 1b) immunohistochemically using the same set of the antibodies used for the resected specimen. The immunophenotype of the rectal biopsy was identical to that of the resected tumor (data not shown). Reexamination and comparison with this biopsy sample revealed that the tumor cells in the resected specimen appeared less cohesive than those in the rectal biopsy. This is probably due to the effect of the chemo-radiation therapy on the tumor cells before surgical resection.

Mammography, ultrasonography, and ^18^F-fluorodeoxyglucose-positron emission tomography (FDG-PET)/computed tomography (CT) examination revealed no evidence of cancer in her remaining right breast and a local recurrence of the left breast cancer (data not shown). There were two hot spots at the rectum and thyroid on FDG-PET/CT examination (data not shown). The former was considered to result from radiation therapy and/or proctectomy procedure, and the latter was presumably due to chronic thyroiditis.

Although we could not reconfirm the diagnosis of the primary breast cancer, the results described above strongly support our final diagnosis of rectal metastasis from lobular breast carcinoma.

## Discussion

In this paper, we reported a case of rectal metastasis from lobular breast carcinoma, masquerading as a primary rectal cancer. The correct diagnosis was obtained by histopathological examination of the resected rectal mass after chemo-radiotherapy.

To our knowledge, at least 21 cases have been reported on metastatic lobular carcinoma of the breast to the colorectum [1-21]. There have been four reviews on the clinical or radiological spectrum of metastatic lobular carcinoma of the breast to the colorectum [22-25]. McLemore *et al*. reported that cases of gastrointestinal metastasis from primary breast cancer were as rare as 73 cases among 12,001 cases [22]. Among them, cases with colorectal metastasis were only 24. As histological subtypes, lobular carcinoma of the breast predominates in comparison to ductal carcinoma [22,23].

In our case, history of primary breast cancer was unknown in the initial presentation of the colorectal lesion; the history was revealed after histopathological examination of the resected specimen. Among the 21 cases reported of metastatic lobular carcinoma of the breast to the colorectum, only one case was similar to our case [1]. In two cases, breast cancer and presumed metastasis were found coincidentally [2,3]. In the majority of the cases, a history of primary breast cancer was known in the initial presentation of gastrointestinal lesions and it was not difficult to suspect those lesions as relapse or metastasis of the breast cancer [4-14]. In one case, history of primary breast cancer was known in the initial presentation of the gastrointestinal lesions, but correct diagnosis could not be reached [15]. Harsløf *et al*. reported a case compatible with metastasis of lobular carcinoma of the breast to the colon although the primary breast cancer had not been identified [16].

There are a couple of reasons for difficulties in distinguishing metastatic lobular carcinoma of the breast to the colorectum from the primary colorectal cancer. First, the interval between the primary breast cancer and its metastatic relapse tends to be long. Therefore, a key to the correct diagnosis is recognition of a patient’s history of breast cancer. Schwarz *et al*. reported that median interval between breast cancer and gastrointestinal metastasis was 6 years (range 0.25 to 12.5 years) [24]. McLemore *et al*. reported that mean interval between breast cancer and gastrointestinal metastasis was 7 years [22]. Among all the cases reported in the literature, 25 years was the longest interval between the diagnosis of the primary breast lobular carcinoma and the recognition of its metastasis to the colorectum [4]. Mistrangelo *et al*. reported a case for sigmoid colon metastasis from the primary lobular carcinoma of the breast 25 years before [4]. However, in that case, there was an episode of bone metastasis of the breast cancer 12 years before the colon metastasis [4]. In contrast, in our case, the 24-year interval was the second longest one and was disease-free. This suggests that a long-term, close follow-up as well as recognition of patients’ history is required for correct diagnosis of rectal metastasis from lobular breast carcinoma.

Secondly, metastatic lobular carcinoma of the breast to the colorectum presents endoscopic and radiologic appearance of linitis plastica-type lesion with circumferential stricture and wall thickening of the colorectum [1,25]. Arrangoiz *et al*. reported a case in which only pediatric colonscope could pass the narrowed lumen of the lesion [1]. Colorectal metastasis of lobular breast carcinoma is sometimes accompanied by nodular and cobble stone-like thickening of mucosa [5]. These macroscopic characteristics will present impression of the lesion as a poorly differentiated carcinoma as is often seen in the stomach. When the metastasis occurs around the terminal ileum lesion, a misdiagnosis may be made as an inflammatory bowel disease as Crohn’s disease [6,7,15]. In fact, Calafat *et al*. reported metastasis of lobular carcinoma of the breast to ileum-colon, mimicking inflammatory bowel disease such as Crohn’s disease [6]. Koos *et al*. reported a case where multiple metastases simulating Crohn's disease were found radiologically and intra-operatively in the colon and small bowel [7]. When metastatic cancer remains in submucosa, it will be difficult to obtain enough biopsy samples endoscopically, further strengthening the impression that the lesion may be non-tumorous [15].

## Conclusions

Poorly differentiated adenocarcinoma of the colorectum is rarer than that of the stomach. Linitis plastica-type cancer of the colorectum is also rarer than that of the stomach. A lesson from the present case is that before we conclude a linitis plastica-type cancer of poorly differentiated type as a primary colorectal cancer, it is critical to exclude the possibility of metastatic colorectal cancer.

## Consent

Written informed consent was obtained from the patient for publication of this Case report and any accompanying images. A copy of the written consent is available for review with the Editor-in-Chief of this journal.

## Abbreviations

CT: Computed tomography; FDG-PET: ^18^F-fluorodeoxyglucose-positron emission tomography; HE: Hematoxylin and eosin.

## Competing interests

The authors declare that they have no competing interests.

## Authors’ contributions

IM and SH participated in the pathological final diagnosis of the case and prepared and edited the manuscript. NA performed the preoperative endoscopic examination. NM, MH, DY, TK, KT, TY, MN, HI, and NT were responsible for the chemo-radiotherapy, operations, and follow-up of the patient and helped IM and SH in preparation of the manuscript. All authors read and approved the final manuscript.
